# Effect of exercise on abdominal obesity and inflammatory response in the older adults: a systematic review and meta-analysis of randomized controlled trials

**DOI:** 10.3389/fspor.2025.1677087

**Published:** 2026-01-06

**Authors:** Yangjun Liu, Sujie Mao, Wei Xie, Guoping Qian, Xia Wu, Hanxiao Xu, Zbigniew Ossowski

**Affiliations:** 1 School of Physical Education and Health, Chengdu University of Traditional Chinese Medicine, Chengdu, Sichuan, China; 2 Faculty of Physical Culture, Gdansk University of Physical Education and Sport, Gdańsk, Poland; 3 Discipline Construction Office, Nanjing Sport University, Nanjing, Jiangsu, China

**Keywords:** exercise, abdominal obesity, inflammatory markers, CRP, TNF-α, IL-6, adiponectin

## Abstract

**Background:**

Abdominal obesity and chronic inflammation are key indicators of aging, associated with various age-related diseases. While exercise is thought to mitigate these issues, its specific effects on abdominal obesity, adiponectin, and inflammatory markers in older adults need further exploration.

**Objective:**

This systematic review and meta-analysis aimed to evaluate the effects of exercise on abdominal obesity, adiponectin, and inflammation markers in older adults.

**Methods:**

A search was conducted up to February 20, 2025, using six electronic databases (Medline, Web of Science, Embase, CINHAL, Scopus, and Cochrane). The inclusion criteria focused on randomized controlled trials assessing exercise interventions in healthy older adults or those with obesity, diabetes, or metabolic syndrome (MetS). Relevant citations were analyzed using Rayyan software, while the quality of studies was assessed with the Cochrane risk of bias tool, and data were meta-analyzed using Review Manager (RevMan) 5.4 software.

**Results:**

From 7,622 citations, 128 articles were screened for full text, resulting in 19 RCTs with 1,130 participants included. The meta-analysis indicated that exercise (aerobic, resistance, or combined training) significantly reduced abdominal obesity, demonstrated by a decrease in waist circumference (WC) with a mean difference (MD) of −2.03 cm (95% confidence interval [CI]: −4.06 to −0.01, *p* = 0.05) and body mass index (BMI) with an MD of −0.49 kg/m^2^ (95% CI: −0.70 to −0.27, *p* < 0.0001). Furthermore, exercise lowered levels of C-reactive protein (CRP, MD = −0.07 mg/L, 95% CI: −0.13 to −0.02, *p* = 0.006), tumor necrosis factor-α (TNF-α, MD = −0.66 pg/mL, 95% CI: −1.07 to −0.25, *p* = 0.002), and interleukin-6 (IL-6, MD = −0.33 pg/mL, 95% CI: −0.60 to −0.05, *p* = 0.02). Key subgroup findings included: (1) Obese older adults and those with MetS experienced 2–3 times greater WC reduction than healthy peers; (2) Vigorous physical activity (VPA) was more effective than moderate-to-vigorous physical activity (MVPA) in lowering TNF-α; (3) Short-term interventions (<6 months) prioritized inflammation reduction (TNF-α, IL-6), while long-term interventions (≥6 months) better improved BMI. No significant changes in adiponectin levels were observed (MD = 0.15 μg/mL, 95% CI: −0.43 to 0.72, *p* = 0.61).

**Conclusion:**

Exercise has a positive effect on abdominal obesity in older adults and reduces levels of several inflammatory markers. Further randomized controlled trials are needed to better understand the effects of exercise on other inflammatory markers.

**Systematic Review Registration:**

https://www.crd.york.ac.uk/PROSPERO/view/CRD42023404011, PROSPERO CRD42023404011.

## 1 Introduction

Physical inactivity in older adults contributes to adverse health outcomes, including central and peripheral obesity and systemic inflammation (1). Abdominal obesity defined by elevated visceral fat area (VFA) and waist circumference (WC) is closely linked to metabolic disorders, hypertension, and cardiovascular disease (CVD) (2), while obesity itself induces chronic inflammation and raises inflammatory marker levels (3). Adipose tissue secretes key inflammatory markers such as C-reactive protein (CRP), interleukin-6 (IL-6), adiponectin, leptin, and TNF-α (4–6); among these, adiponectin inversely correlates with VFA accumulation, protects against obesity related diseases, and attenuates CRP/TNF-α expression in adipose tissue—though its production is suppressed by TNF-α and IL-6 (7, 8).

In older adults, CRP independently predicts CVD onset (9–14), while IL-6 and TNF-α act as health risk factors and stimulate hepatic CRP release (15, 16). These markers are associated with increased risk of CVD, diabetes, cancer, and disability (11, 14, 17–22), and chronic inflammation drives age-related diseases (e.g., CVD, cancer, diabetes, Alzheimer's disease) and predicts mortality in healthy older adults (23–26). Elevated circulating inflammatory mediators are common in age-related diseases (27). Physical inactivity, which is prevalent in older adults, exacerbates obesity (especially abdominal obesity) and chronic inflammation (28, 29). Exercise emerges as a promising strategy to prevent obesity and improve inflammatory responses, potentially preceding pharmacological interventions for age-related chronic diseases (30, 31).

Prior meta-analyses on exercise's effects in older adults show inconsistent conclusions. Sardeli et al. (32) found resistance training (RT) reduced CRP (SMD = −0.61, *p* < 0.001) but not TNF-α in adults over 50. Zhuang et al. (33) reported no exercise effect on inflammatory markers in older adults with sarcopenic obesity. Liang et al. (34) highlighted combined exercise's superiority for metabolic parameters, while Zheng et al. (35) showed aerobic exercise lowered CRP, TNF-α, and IL-6 in healthy middle-aged/older adults. These discrepancies—due to varied populations, exercise types, and outcomes—limit evidence clarity. Thus, our meta-analysis is needed to unify findings via standardized subgroup analyses (intensity, duration, comorbidities) (35, 36).

## 2 Methods

This meta-analysis is reported following the PRISMA (Preferred Reporting Items for Systematic Reviews and Meta-Analyses) guidelines (37). The review was registered in International prospective register of systematic reviews, PROSPERO protocol CRD42023404011.

### 2.1 Literature search strategy

The databases Medline (via PubMed), Web of Science, Embase, CINHAL, Scopus, and the Cochrane Library were searched to find pertinent studies. The literature search was performed according to the PICOS strategy, as follows: (P) Population: Older adults aged 60 years and above, (Studies calculated in terms of mean age were also included), regardless of gender or health status (healthy or with chronic conditions); (I) Intervention: exercise; (C) Comparator: exercise intervention group and non-exercise control group; (O) Outcomes: Obesity and Inflammation marker levels; and (S) Study type: RCTs. Exercise, training, RT, strength training, and high-intensity interval training with older adults, elderly, aged, elder and obese with inflammation, cytokines, inflammation, and interleukin binding were the major keywords used to find pertinent literature. We included English-language RCTs that met the criteria published from the date when the database was created until February 2025. A complete list of the search algorithms, terms, and results can be found in Supplementary Table S1.

### 2.2 Selection and exclusion criteria of the literature

YJ. L and SJ. M, searched the databases of the included studies at the same time. The article selection process involved importing articles from all databases into Rayyan software (38) to identify and remove duplicate documents. The screening process consisted of two phases: summary filtering and full-text filtering. According to the PROSPERO protocol CRD42023404011, four examiners (YJ. L, YM. L, J.Y, X.W) independently reviewed the selected literature to determine study eligibility. In case of disagreement, the reviewer (O.Z.) was consulted. Data collection and collation were performed by two reviewers (YJ. L and SJ.M). The literature included in the analysis was limited to randomized controlled trials (RCTs) that were peer-reviewed publications and focused on the biological effects of exercise interventions in older adults. The inclusion criteria did not specify gender and included both healthy individuals and those with chronic conditions, as long as they were over 60 years of average age.

### 2.3 Data extraction and quality assessment

The following are the main research characteristics: (A) Participants: Age, type of disease and sample size, etc. (B) Exercise: type, frequency, duration, and intensity. (C) Outcome measurements: Obesity indicators include WC, body mass index (BMI), body fat percentage (BF%), and VFA. It is explicitly clarified that “abdominal circumference” analyzed in this study corresponds to WC in the original studies, which refers to the midpoint between the lower rib and the top of the iliac crest measured at the level of the umbilicus (navel); circumference data measured at the hip or other abdominal sites are excluded. Inflammatory markers: Biomarker data (e.g., CRP, TNF-α, IL-6, adiponectin) were obtained in accordance with the standard operating procedures of the respective laboratories in the original studies.

Two authors (YJ.L and SJ.M) independently assessed the risk of bias in the included studies using the Cochrane Risk of Bias Instrument for randomized controlled trials (RoB2) (39), which includes six different domains as the following: (A) Bias arising from the randomisation process; (B) Bias due to deviations from intended interventions; (C) Bias due to missing outcome data; (D) Bias in measurement of the outcome; (E) Bias in selection of the reported result; (F) Overall bias. In case of any discrepancies, a third reviewer (O.Z) was consulted to resolve them.

### 2.4 Data synthesis and statistical analyses

For each relevant outcome, means and standard deviations (SDs) before and after intervention were extracted and used to generate forest plots via meta-analysis. When means and SDs were not reported, data were estimated from standard errors, medians, ranges, or interquartile ranges as per established methods (40–42). Data extraction from figures was performed using Getdata Graph Digitizer software when necessary. All studies with two-arm exercise interventions were included; for multi-arm studies, only data comparing exercise and non-exercise control groups were incorporated. Analyses were conducted using RevMan version 5.4, calculating standardized mean differences (SMDs) with 95% confidence intervals (CIs) under a random-effects model. Effect sizes compared the impact of exercise vs. control on inflammatory markers. Heterogeneity was assessed with Cochran's Q statistic, with *p* < 0.1 indicating significant heterogeneity (43). Subgroup analyses explored the effects of exercise across different disease populations. Publication bias was evaluated both visually via funnel plots and statistically using Egger's test. Sensitivity analyses tested the robustness of pooled results by excluding studies with a high risk of bias.

Given the expected variability in intervention form, intensity, and duration likely contributing to high heterogeneity a random-effects model was employed. Subgroup and sensitivity analyses focused on primary outcomes such as WC, BMI, and inflammatory markers (CRP, TNF-α) to identify sources of heterogeneity and strengthen confidence in the results. Intervention intensities were categorized into MVPA and VPA based on the Physical Activity Metabolic Equivalence Guidelines, considering reported exercise intensity, heart rate ranges, and training modalities. Intervention types (aerobic, resistance, or combined training) and durations were classified according to their core components and original descriptions in the included studies.

## 3 Results

### 3.1 Study selection and search results

**Figure 1:**
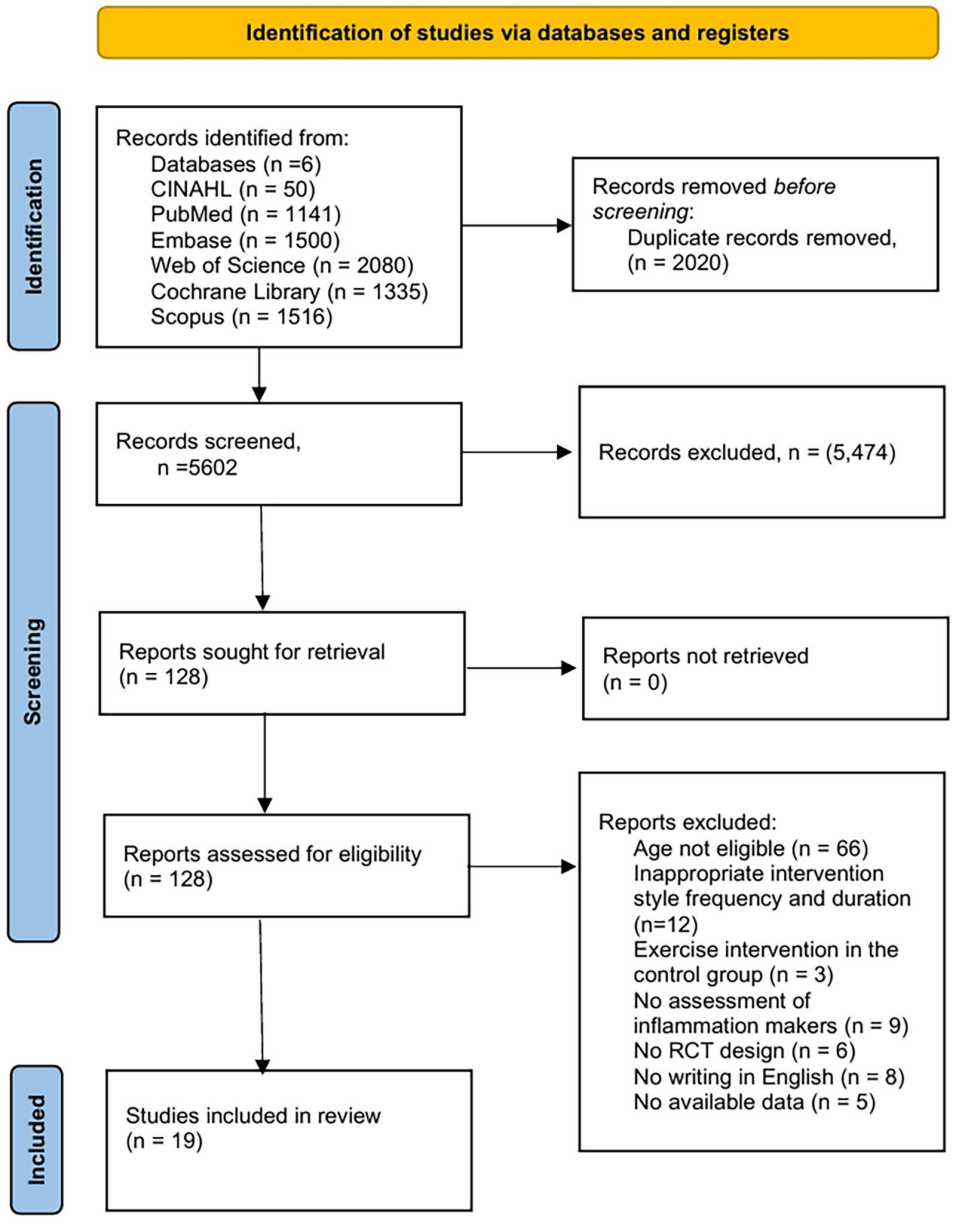
Flowchart for searching and screening of the included studies.

A total of 7,622 records were identified through the systematic search across six electronic databases. The number of records retrieved from each database was as follows: Medline (via PubMed) (*n* = 1,141), Web of Science (*n* = 2,080), Embase (*n* = 1,500), CINAHL (*n* = 50), Scopus (*n* = 1,516), and the Cochrane Library (*n* = 1,335). After removing 2,020 duplicate records, four reviewers (YJ. L, YM. L, J.Y, X.W) independently screened the remaining 5,602 records by title and abstract, excluding 5,474 irrelevant studies. Subsequently, 128 articles were selected for full-text eligibility assessment. Following rigorous evaluation against the inclusion and exclusion criteria, 19 studies were ultimately included in this meta-analysis. The detailed study selection process is illustrated in Figure 1, which presents a flowchart depicting the identification, screening, and inclusion of eligible studies.

### 3.2 Characteristics of included studies

Table 1 presents the detailed characteristics of the nineteen included RCTs (1,130 participants). Regarding participant demographics: seventeen studies reported gender (710 women, 307 men) and two studies (113 participants) had unknown gender; the cohort included 177 adults with diabetes (three studies), 567 overweight/obese adults (eight studies), 261 adults with MetS (five studies), and 125 healthy older adults (three studies).

**Table 1:** Characteristics of the included studies.

First author, Year/Country	Sample size n(F/M)	Mean age (SD)	Chronic condition	Intervention	FITT principle	Control group(s)	Outcomes
Takahashi M; 2013/Japan	E: 14 (0)	67.8 (1.3)	0	Aerobic exercise (walking)	2 times/week; 12 weeks; 30–60 min/session.	Maintain their lifestyle, including dietary habits.	WC
							BMI
	C: 14 (0)	71.4 (1.6)					BF%
							TNF-α
							IL-1b
							IL-10
Mendoza-Núñez VM; 2018/Mexico	E: 48 (0)	67.4 (4.7)	MetS	Aerobic exercise (Tai Chi)	5 times/week; 6 months.	Without physical exercise practice	BMI
	C: 37 (0)	68.2 (6.6)					CRP
							TNF-α
							IL-6
							IL-8
							IL-10
Beavers KM; 2013/America	E: 97	67.0 ± 4.8	Overweight or Obese	Aerobic exercise (walking and interactive)	3–5 times/week, 150 min/week, 18 months, 13 on the Borg Scale.	Successful aging	Adiponectin
	C: 93						Leptin
	Total (63/127)						IL-6
							IL-8
							TNF-α
Bouchonville M; 2013/America	E: 26 (16/10)	70 (4)	Obese	Multicomponent exercise sessions	3 times/week; 12 months; 65–85%peak heart rate.	Without physical exercise practice	WC
	C: 27 (18/9)	69 (4)					BMI
							VF
							Adiponectin
							TNF
							CRP
Gargallo P; 2024/Spain	E1: 25 (250/0)	69.30 (4.96)	MetS	Multicomponent training or Power training	2 times/week; 20 weeks, scale (Borg, 1998) 11 and 15.	Maintain their life and initial nutritional habits	WC
	E2: 22 (22/0)	67.24 (4.42)					BMI
	C: 25 (25/0)	68.00 (5.01)					BF%
							CRP
Mavros Y; 2014/Australia	E: 41 (19/22)	67.2 (4.9)	Diabetes	Progressive resistance training	3 times/week; 12 months; 80%(1RM) or Borg scale rating 15 and 18	The sham-exercise group trained 3 times a week Light exercise.	BMI
	C: 47 (23/24)	69.2 (6.3)					VAT
							CRP
Sardeli AV; 2022/Brazil	E: 12 (12/0)	64.5 (3.9)	MetS	Resistance and Aerobic exercise	3 times/week; 55 min/session, 16 weeks; 63% maximum VO2max.	Maintain their usual physical activity	WC
	C: 13 (13/0)	65.6 (5.6)					BMI
							BF%
							TNF-α
							CRP
Kadoglou NP; 2007/Greece	E: 30 (17/13)	59.33 (4.76)	Diabetes	Aerobic exercise (walking or running or, cycling)	4 times/week; 6 months;60 min (50–75%, VO2 peak)	Control patients were instructed to maintain their habitual activities	BMI
	C: 30 (18/12)	63.82 (7.03)					BF%
							CRP
							TNF-a
							Adiponectin
							IL-10
							IL-18
Christensen P; 2013/Denmark	E: 64 (52/12)	62.9 (5.8)	Obese	Functional weight-bearing exercises	4 times/week; 52 weeks; 60 min; 4–6 MET	Served as a usual-care	WC
	C: 64 (51/13)	61.7 (6.8)					BMI
							CRP
Miller EG; 2017/Australia	E: 16 (6/10)	67.6 (5.2)	Diabetes	Resistance training	3 times/week; 6 months; 75%–85% maximum strength 1-RM.	Stationary cycling with no workload for 5 min followed by a series of static stretching exercises	BMI
	C: 13 (7/6)	66.9 (5.3)					IL-6
							IL-10
							TNF-α
							Adiponectin
Hasegawa N; 2017/Japan	E: 26 (13/13)	68.0 (7.0)	0	Aerobic exercise training	3 times/week; 8 weeks; 40%–70% VO2 peak.	Not to change their level of physical activity	BMI
	C: 26 (13/13)	65.8 (8.6)					VFA
							Adiponectin
							CRP
Tomeleri CM; 2017/Brazil	E: 22 (22/0)	72.1 (6.3)	0	Resistance training	3 times/week; 12 weeks; 10–15 RM	Without physical exercise practice	WC
	C: 23 (23/0)	68.8 (4.9)					BMI
							BF%
							CRP
							TNF-α
							IL-6
Da Silva MAR; 2020/Portugal	E1: 13 (11/2)	71.1 (4.8)	MetS	Resistance training + Moderate-intensity continuous training	3 times/week; 50 min/session, 12 weeks; 10–15 RM	Without physical exercise practice	WC
	E2: 13 (9/4)	63.3 (7.2)					BMI
	C: 13 (9/4)	67.4 (3.9)					FM
							CRP
Ahn N; 2022/Korea	E: 20 (20/0)	68.23 (2.56)	MetS	Dynamic-resistance exercise	3 times/week; 40 min/session, 6 months;	Without physical exercise practice	WC
	C: 20 (20/0)	71.42 (5.87)					CRP
							IL-4
							IL-5
							IL-6
							TNF–α
Son WH; 2023/Korea	E: 14 (14/0)	70.2 (1.21)	Obese	Aerobic exercise (walking)	1–7 times/week, 12 weeks, 64–76% HRmax	Without physical exercise practice	BMI
	C: 12 (12/0)	69.9 (1.14)					BF%
							IL-6
							TNF-α
							CRP
Cai Y; 2023/China	E: 17	63.41 (5.06)	Overweight or Obese	Yijinjing and Resistance exercise	3–5 times/week; 68–76 min/session, 6 months, 60%–70% HRmax	Without physical exercise practice	BMI
	C: 17	61.82 (4.33)					FM
							BF%
							IL-6
							TNF-α
	Total (21/13)						
Chagas EFB; 2017/Brazil	E: 35 (35/0)	61.3 (6.4)	Obese	Aerobic exercise (walking) and Neuromuscular Training	3 times/week; 75 min/session, 20 weeks; 50%–60% of VO2peak.	Without physical exercise practice	WC
	C: 35 (35/0)	59.8 (7.1)					BMI
							BF%
							IL-6
							TNF-α
Tomeleri CM; 2016/Brazil	E: 19 (19/0)	66.8 (3.2)	Obese	Resistance training	3 times/week, 45–50 min/session, 8 weeks, 50–100% RM.	Did not perform any type of physical exercise	BMI
	C: 19 (19/0)	69.5 (4.7)					BF%
							IL-6
							TNF-α
							CRP
Azamian Jazi A; 2022/Iran	E: 14 (14/0)	73.29 (5.44)	Overweight	Elastic bands resistance training	3 times/week; 55 min/session, 12 weeks; 12–14 of the Borg scale	Without physical exercise practice	BMI
	C: 14 (14/0)	74.79 (3.87)				
							BF%
							TNF-α
							CRP

F, female; M, male; SD, standard deviation; FITT, frequency-interval-time-type principle; E, experimental group; C, control group; RM, repetition maximum; MetS, metabolic syndrome; WC, waist circumference; BMI, body mass index; CRP, C-reactive protein; TNF-α, tumor necrosis factor alpha; IL-4/5/6/8/10, interleukin-4/5/6/8/10; BF%, body fat percentage; VF, visceral fat; VFA, Visceral fat area; VAT, visceral adipose tissue area; FM, fat mass.

Exercise interventions varied by type: six studies used aerobic exercise (walking, running, cycling), seven focused on RT, three combined aerobic and RT, two used progressive RT, and one each used tai chi, Yi Jinjing plus RT, and circuit training. Intervention duration ranged from 8 weeks to 18 months, with 2–5 sessions per week and 30–90 min per session.

Exercise intensity classification was based on individual exercise intensity (not “intensity of classes”) and referenced two standardized criteria: the Compendium of Physical Activities (44, 45) and the Body Activity Exercise Rating Assessment DELPHI Scale. Specific division principles and intensity thresholds are as follows: MVPA: Defined as 3.0–5.9 metabolic equivalents (METs), including activities such as brisk walking (3.5–5.9 METs), moderate-intensity cycling (4.0–5.5 METs), and light RT (3.0–5.0 METs). VPA: Defined as ≥6.0 METs, including activities such as jogging (6.0–8.9 METs), high-intensity interval training (≥9.0 METs), and high-intensity RT (≥6.0 METs). For studies that did not directly report MET values, intensity was converted using objective or subjective indicators from the original literature: Heart rate (HR): HR 60%–75% of maximum heart rate (220—age) was mapped to MVPA; HR >75% of maximum heart rate was mapped to VPA. Subjective Perceived Exertion Rating of Perceived Exertion (RPE): RPE 12–14 (on the 6–20 Borg Scale) was mapped to MVPA; RPE 15–17 was mapped to VPA. Based on this classification, 9 studies were categorized into the MVPA group and 10 into the VPA group. Detailed MET values (including single-session METs and weekly total MET-minutes) for each intervention are provided in Supplementary Table S2.

### 3.3 Risk of bias of the included studies

**Figure 2:**
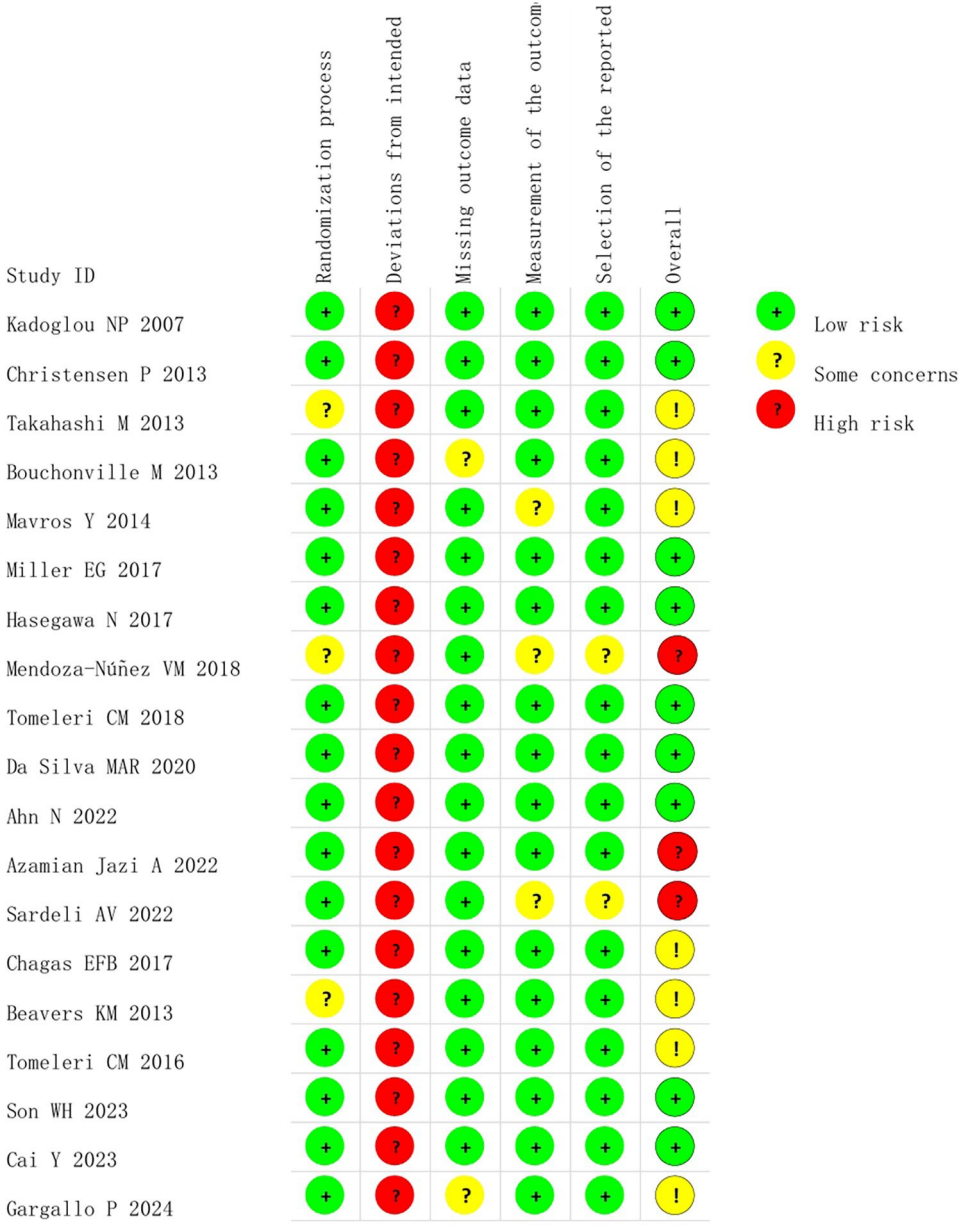
Risk of bias in included studies: review authors' judgements about each risk of bias item for each included study.

**Figure 3:**
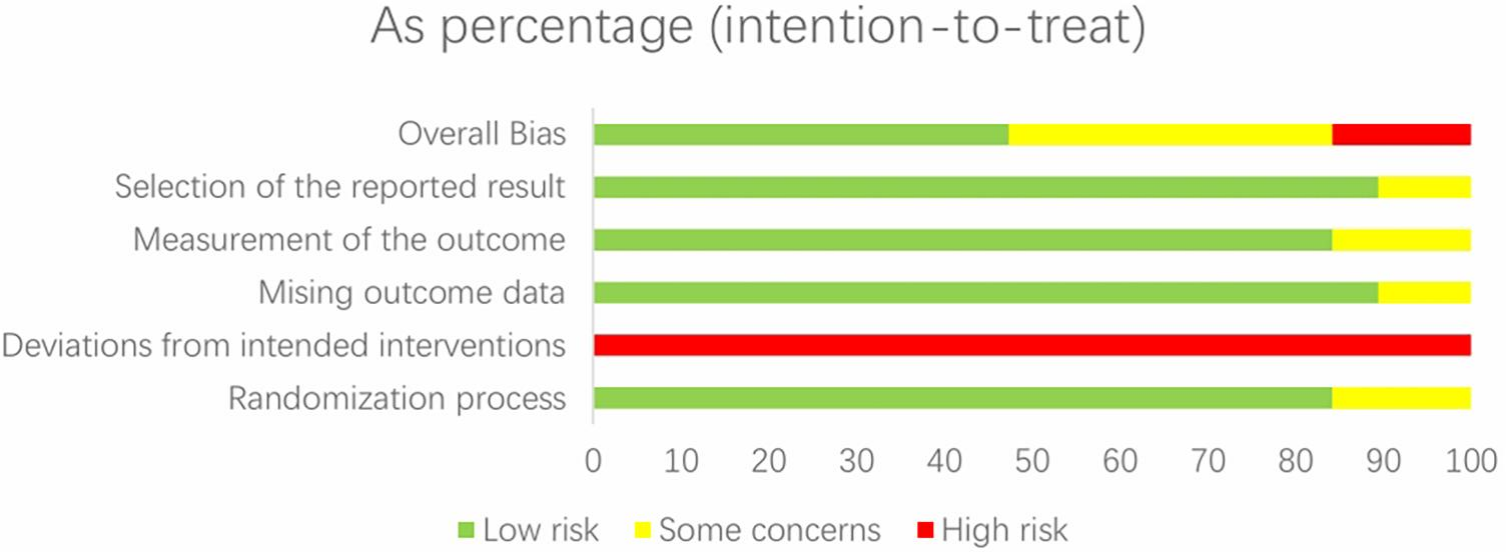
Risk of bias graph: review authors' judgements about each risk of bias item presented as percentages across all included studies.

Randomization was reported in all included articles, but three trials (46–48) did not describe the specific method of randomization. Blinding of participants and personnel is difficult since the included experiments are all human studies; the participants need to sign an informed consent form, and the relevant researchers need to supervise during exercise interventions. Therefore, the item has been evaluated as high risk in all current studies. Two studies (49, 50) were at risk of missing outcome data; Three studies (47, 51, 52) may have bias in measurement of the outcome; Two studies (47, 52) were biased in selection of the reported result. Nine studies (52–60) were rated as low risk overall; Seven studies (46, 48–51, 61, 62) were rated as moderate risk overall; Two studies (47, 63) were rated as high risk overall. The risk of bias assessment of the included studies is shown in Figures 2, 3.

### 3.4 Effect of interventions

Central obesity (abdominal adiposity) is a key risk factor for noncommunicable diseases, with WC as the primary indicator of abdominal obesity; BMI, BF% and VFA as a secondary obesity measure. For inflammatory markers, we focused on those reported in ≥three studies (CRP, TNF-α, adiponectin, IL-6); leptin and IL-4/5/10/18 were excluded due to insufficient data. Units of adiponectin (mg/mL, μg/mL, ng/mL) and CRP (mg/dL, mg/Lb, mg/L, pg/mL) were standardized to μg/mL and mg/dL, respectively. For 2 studies with two intervention groups (50, 57), groups were combined per the Cochrane Manual (Chapter 7, Section 7.7.a) for analysis. Dietary data were unavailable across all included studies, so the observed effects of exercise on obesity and inflammatory markers may not account for potential dietary confounders when introducing outcome analyses.

### 3.5 Outcome measures

The effect of the intervention on WC was evaluated in a total of eight studies (46, 49, 50, 52, 53, 56–58), and a total of eleven studies reported BMI values (46, 47, 52, 53, 55–57, 59–61, 63). Nine studies measured BF% via bioelectrical impedance or dual-energy x-ray absorptiometry (DXA) (50, 52, 56, 59–64), and four studies measured VFA (51, 54, 55, 57), the direct indicator of abdominal fat, via computed tomography. The effect of the intervention on CRP was evaluated in thirteen studies (47, 49–53, 56–59, 62, 63), but one study (58) was excluded from the analysis due to unavailable values. TNF-α was evaluated in fourteen studies (46–49, 52, 54, 56, 58–64), but two studies (48, 49), were unavailable for the meta-analysis due to missing data. Adiponectin was evaluated in four studies (48, 54, 55, 64), and the effects of interventions on IL-6 were evaluated in nine studies (47, 48, 54, 56, 58–62). Leptin and IL-4/5/10/18 were not included in the meta-analysis as they were reported in fewer than three studies.

#### 3.5.1 Waist circumference (WC, cm)

**Figure 4:**
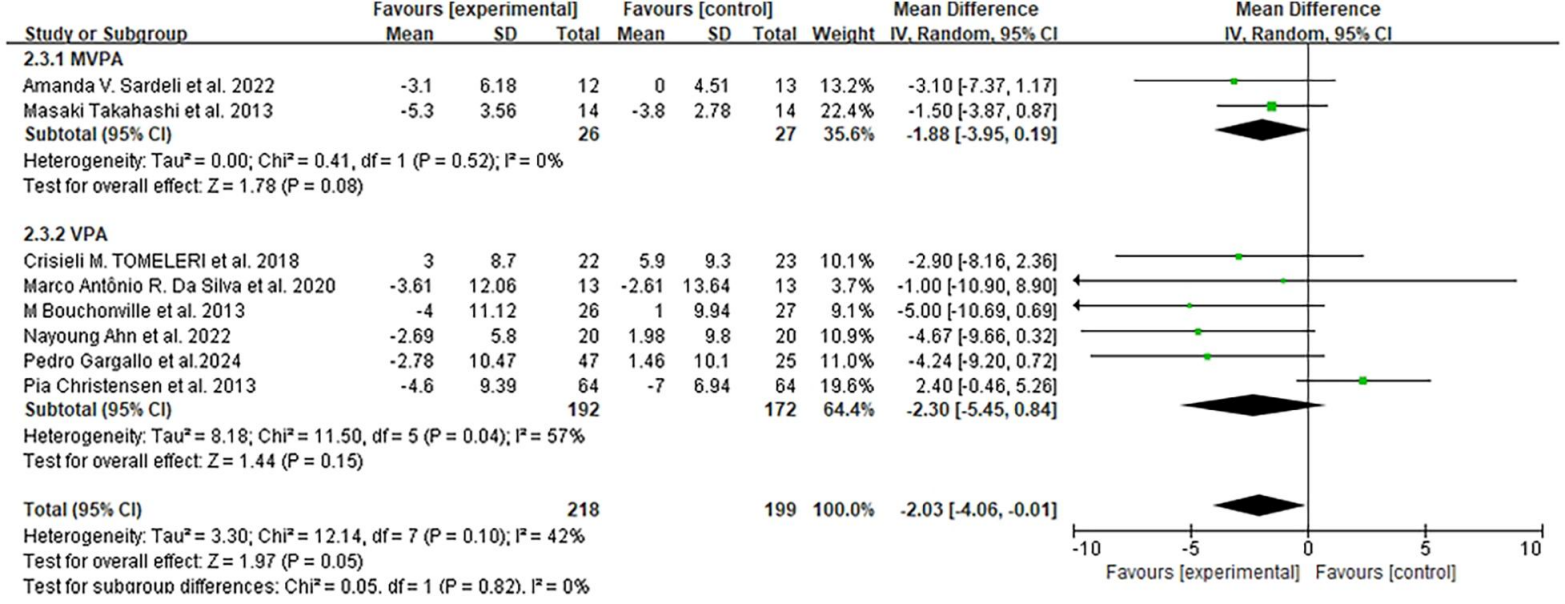
Forest plots of the effect of physical activity on WC compared with no exercise.

Eight studies (417 participants) measured WC at different time points (12 weeks to 36 months) (46, 49, 50, 52, 53, 56–58). Meta-analysis results showed that exercise significantly reduced WC in older adults (MD = −2.03 cm, 95% CI: −4.06 to −0.01 cm, *I*^2^ = 42%, *p* = 0.05), with details shown in Figure 4.

Subgroup analysis by exercise intensity indicated that VPA (six studies) led to a greater WC reduction (2.30 cm) than MVPA (two studies, 1.88 cm). By intervention duration: studies with <6 months of exercise [108 experimental vs. 88 control participants (46, 50, 52, 56, 57)] showed an average WC reduction of 2.26 cm (MD = −2.26 cm, 95% CI: −4.03 to −0.50), while those with ≥6 months [110 experimental vs. 111 control participants (49, 53, 58)] showed a reduction of 2.03 cm (MD = −2.03 cm, 95% CI: −7.46 to 3.41). By exercise type: combined training [aerobic and resistance (49, 50, 52, 57)] had the largest WC reduction 3.71 cm (MD = −3.71 cm, 95% CI: −6.42 to −1.01), followed by aerobic exercise reduction of 1.5 cm (46) and RT reduction of 1.34 cm (56).

By participant health status: exercise had the most significant WC-lowering effect in obese older adults [two studies (49, 53), average reduction 5 cm, MD = −5.00 cm, 95% CI: −10.69 to −0.69], followed by those with MetS [four studies (50, 52, 57, 58), reduction 3.70 cm, MD = −3.70 cm, 95% CI: −6.32 to −0.42]; healthy older adults [two studies (46, 56)] had a minor reduction, MD = −1.74 cm, 95% CI: −3.89 to 0.42).

#### 3.5.2 BMI (kg/m^2^)

**Figure 5:**
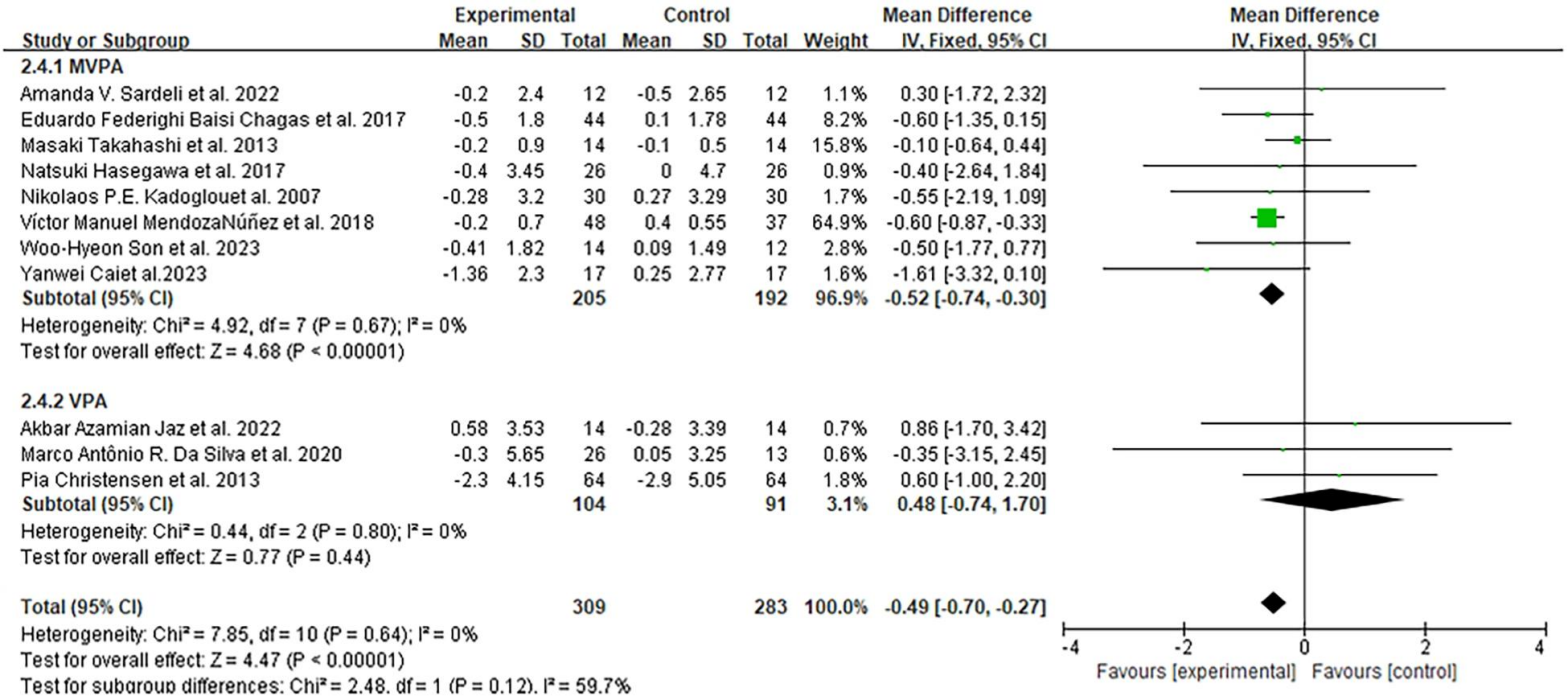
Forest plots of the effect of physical activity on BMI compared with no exercise.

Eleven studies (592 participants) assessed BMI at various time points (8 weeks to 1 year) (46, 47, 52, 53, 55–57, 59–61, 63, 64). Meta-analysis confirmed that exercise significantly reduced BMI in older adults (MD = −0.49 kg/m^2^, 95% CI: −0.70 to −0.27, *I*^2^ = 0%, *p* < 0.0001), with detailed data presented in Figure 5.

Subgroup analysis by exercise intensity showed that MVPA (eight studies) reduced BMI by 0.52 kg/m^2^, slightly more than VPA (three studies, 0.48). By intervention duration: five studies with <6 months of exercise [150 experimental vs. 135 control participants (46, 50, 52, 56, 57)] reported an average BMI reduction of 0.25 kg/m^2^ (MD = −0.25 kg/m^2^, 95% CI: −0.64 to 0.14), while three studies with ≥6 months [159 experimental vs. 148 control participants (49, 53, 58)] showed a more significant reduction of 0.59 kg/m^2^ (MD = −0.59 kg/m^2^, 95% CI: −0.85 to −0.33). By exercise type: combined training [three studies (52, 57, 60)] had the largest BMI reduction of 0.72 kg/m^2^ (MD = 0.72 kg/m^2^, 95% CI: −1.93 to 0.49), followed by RT [two studies (53, 63), MD = −0.67 kg/m^2^, 95% CI: −0.69 to 2.03] and aerobic exercise [five studies (46, 47, 55, 59, 64), 0.51, MD = −0.51 kg/m^2^, 95% CI: −0.73 to −0.29].

By participant health status: three studies on older adults with MetS (47, 52, 57) showed the most notable BMI reduction of 0.58 kg/m^2^ (MD = −0.58 kg/m^2^, 95% CI: −0.84 to 0.41); one study on adults with diabetes (64) reported a 0.55 kg/m^2^ reduction; five studies on obese adults (53, 59–61, 63) showed a reduction of 0.45 kg/m^2^ (MD = −0.45 kg/m^2^, 95% CI: −1.08 to 0.17); and two studies on healthy older adults (46, 59) had a minor reduction of 0.12 kg/m^2^ (MD = −0.12 kg/m^2^, 95% CI: −0.64 to 0.41).

#### 3.5.3 Other obesity indicators

A total of fourteen studies assessed the levels of BF% (%) and VFA (cm^2^) in participants at different time points. Among them, nine studies reported the effect of exercise on participants' BF%: seven studies showed that exercise significantly reduced BF% in the experimental group (50, 56, 59–63), while two studies (52, 64) found no significant reduction in BF% in the experimental group. Additionally, two studies indicated that exercise had no significant effect on participants' fat mass (54, 57). Three studies reported the effect of exercise on VFA: two studies demonstrated that exercise significantly reduced VFA (49, 55), and one study showed no significant effect of exercise on VFA.

#### 3.5.4 CRP (mg/L)

**Figure 6:**
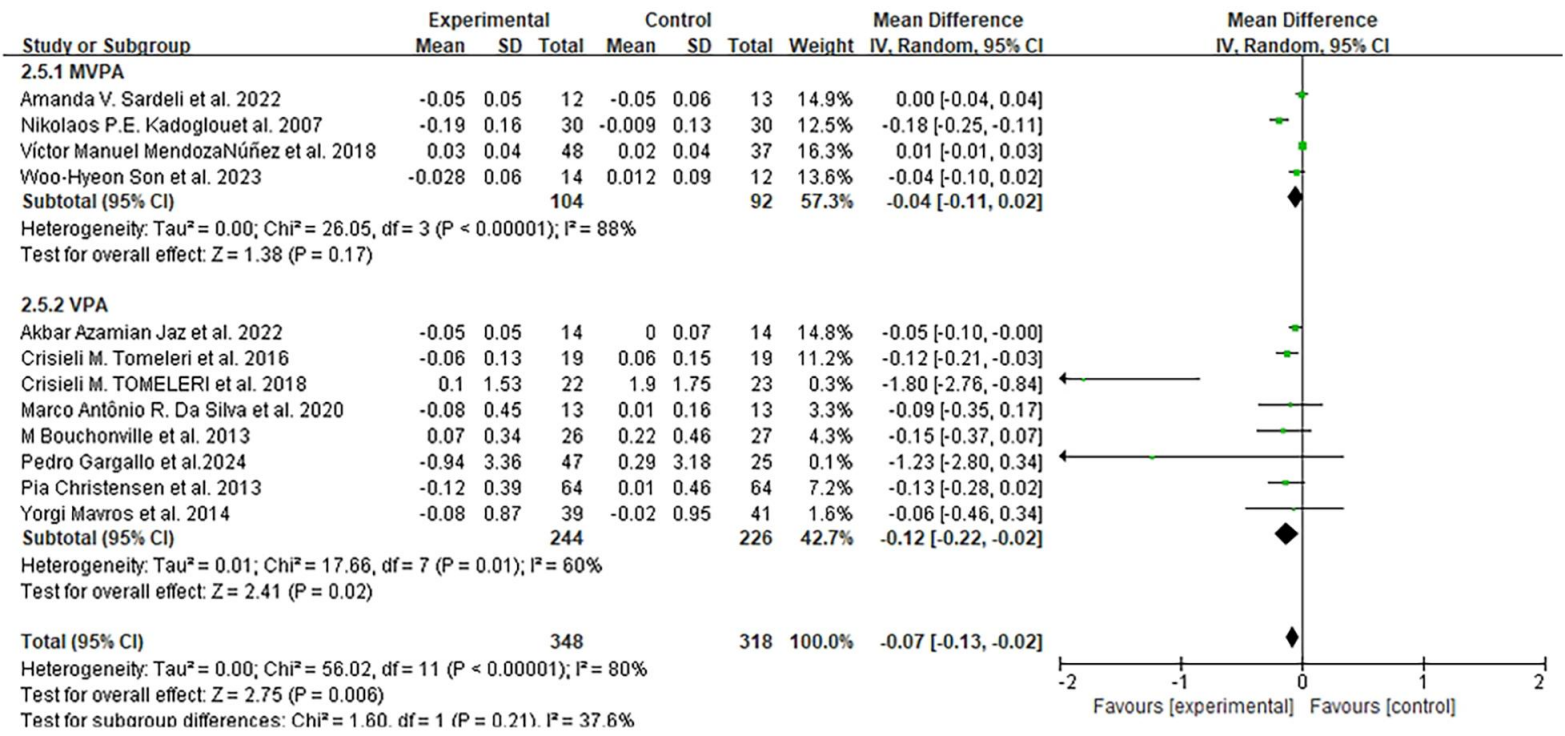
Forest plots of the effect of physical activity on CRP compared with no exercise.

Thirteen studies assessed CRP levels at different intervention durations (8 weeks to 1 year) (47, 49–53, 56–59, 62–64). After excluding one study (58) due to missing data, meta-analysis of twelve studies showed that the exercise group had a significant reduction in CRP compared to the control group (MD = −0.07 mg/L, 95% CI: −0.13 to −0.02, *I*^2^ = 80%, *p* = 0.006), with details presented in Figure 6.

Subgroup analysis by exercise intensity indicated that VPA (eight studies) reduced CRP by 0.12 mg/L, which was more significant than MVPA (four studies, 0.04 mg/L). By intervention duration: seven studies with <6 months of exercise (50, 52, 56, 57, 59, 62, 63) reported an average CRP reduction of 0.06 mg/L (MD = −0.06 mg/L, 95% CI: −0.13 to 0.01), while five studies with ≥6 months (47, 49, 51, 53, 64) showed a reduction of 0.1 mg/L (MD = −0.1 mg/L, 95% CI: −0.22 to 0.02). By exercise type: RT [five studies (51, 53, 56, 62, 63)] had the largest CRP reduction of 0.12 mg/L (MD = −0.12 mg/L, 95% CI: −0.24 to 0.00), followed by aerobic exercise [three studies (47, 59, 64), MD = −0.07 mg/L, 95% CI: −0.17 to 0.04] and combined training [four studies (49, 50, 52, 57), MD = −0.05 mg/L, 95% CI: −0.17 to 0.07].

By participant health status: one study on healthy older adults (56) showed the most significant CRP reduction of 1.8 mg/L (MD = −1.8 mg/L, 95% CI: −2.76 to −0.84); two studies on patients with diabetes (51, 64) reported a reduction of 0.18 mg/L (MD = −0.18 mg/L, 95% CI: −0.25 to −0.10); five studies on obese adults (49, 53, 59, 62, 63) showed a modest reduction of 0.06 mg/L (MD = −0.06 mg/L, 95% CI: −0.09 to −0.03); and four studies on elderly patients with MetS (47, 50, 52, 57) had a minimal reduction of 0.01 mg/L (MD = −0.01 mg/L, 95% CI: −0.01 to 0.03).

#### 3.5.5 TNF-α (pg/mL)

**Figure 7:**
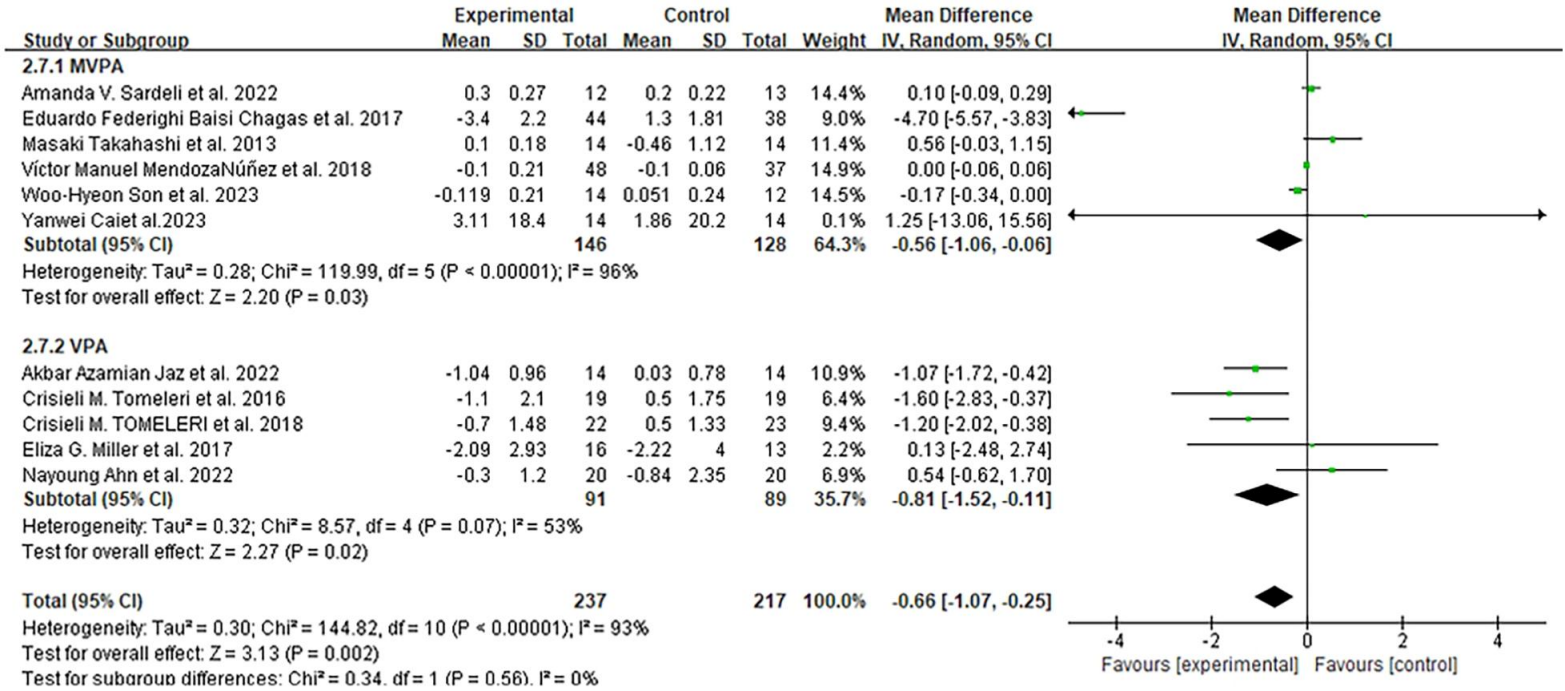
Forest plots of the effect of physical activity on TNF-α compared with no exercise.

Fourteen studies assessed TNF-α levels at various time points (8 weeks to 1 year) (46–49, 52, 54, 56, 58–64). After excluding two studies (48, 49) due to missing data and one study (64) for poor data quality, meta-analysis of eleven studies (454 participants) showed that exercise significantly reduced TNF-α levels compared to the control group (MD = −0.66 pg/mL, 95% CI: −1.07 to −0.25, *I*^2^ = 93%, *p* = 0.002), with details shown in Figure 7.

Subgroup analysis by exercise intensity revealed that VPA (five studies) reduced TNF-α by 0.81 pg/mL (MD = −0.81 pg/mL, 95% CI: −1.52 to −0.11), which was greater than MVPA (six studies, MD = −0.56 pg/mL, 95% CI: −1.06 to −0.06). By intervention duration: eight studies with <6 months of exercise (46, 52, 56, 59–63) demonstrated a significant TNF-α reduction of 1.04 pg/mL (MD = −1.04 pg/mL, 95% CI: −1.76 to −0.33), while three studies with ≥6 months (47, 54, 58) showed no significant change in TNF-α levels between groups. By exercise type: five aerobic exercise studies (46, 47, 59, 61, 64) reduced TNF-α by 1.32 pg/mL (MD = −1.32 pg/mL, 95% CI: −2.76 to 0.11); five RT studies (54, 56, 58, 62, 63) led to a reduction of 0.81 pg/mL (MD = −0.81 pg/mL, 95% CI: −1.52 to −0.11); and two combined training studies (52, 60) showed a slight non-significant increase of 0.10 pg/mL (MD = 0.10 pg/mL 95% CI: −0.09 to 0.29).

By participant health status: five studies on obese older adults (59–63) reported a significant TNF-α reduction (MD = −1.81 pg/mL, 95% CI: −3.65 to 0.02), while no significant effects were observed in older adults with diabetes mellitus, MetS, or healthy populations. Sensitivity analysis (excluding studies with missing data or high bias) reduced heterogeneity but maintained consistent effect direction.

#### 3.5.6 Adiponectin (µg/mL)

**Figure 8:**
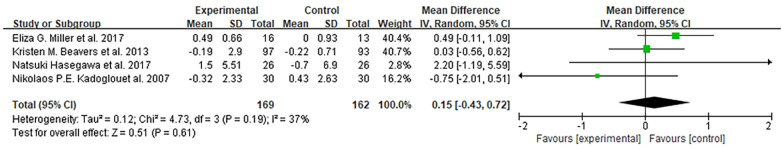
Forest plots of the effect of physical activity on adiponectin compared with no exercise.

Four studies (8 weeks to 18 months) (48, 54, 55, 64) assessed adiponectin levels in older adults. Meta-analysis showed that exercise had no significant effect on adiponectin levels (MD = 0.15 µg/mL, 95% CI: −0.43 to 0.72, *I*^2^ = 37%, *p* = 0.61), with detailed data in Figure 8.

Subgroup analysis by exercise modality indicated inconsistent results: three aerobic exercise studies (48, 55, 64) reported an average adiponectin reduction of 0.08 µg/mL (MD = -0.08 µg/mL, 95% CI: −0.94 to 0.78), while one RT study (54) observed a non-significant increase of 0.49 µg/mL (95% CI: −0.11 to 1.09). Overall, exercise exerted minimal or inconsistent effects on adiponectin levels in this population.

#### 3.5.7 IL-6 (pg/mL)

**Figure 9:**
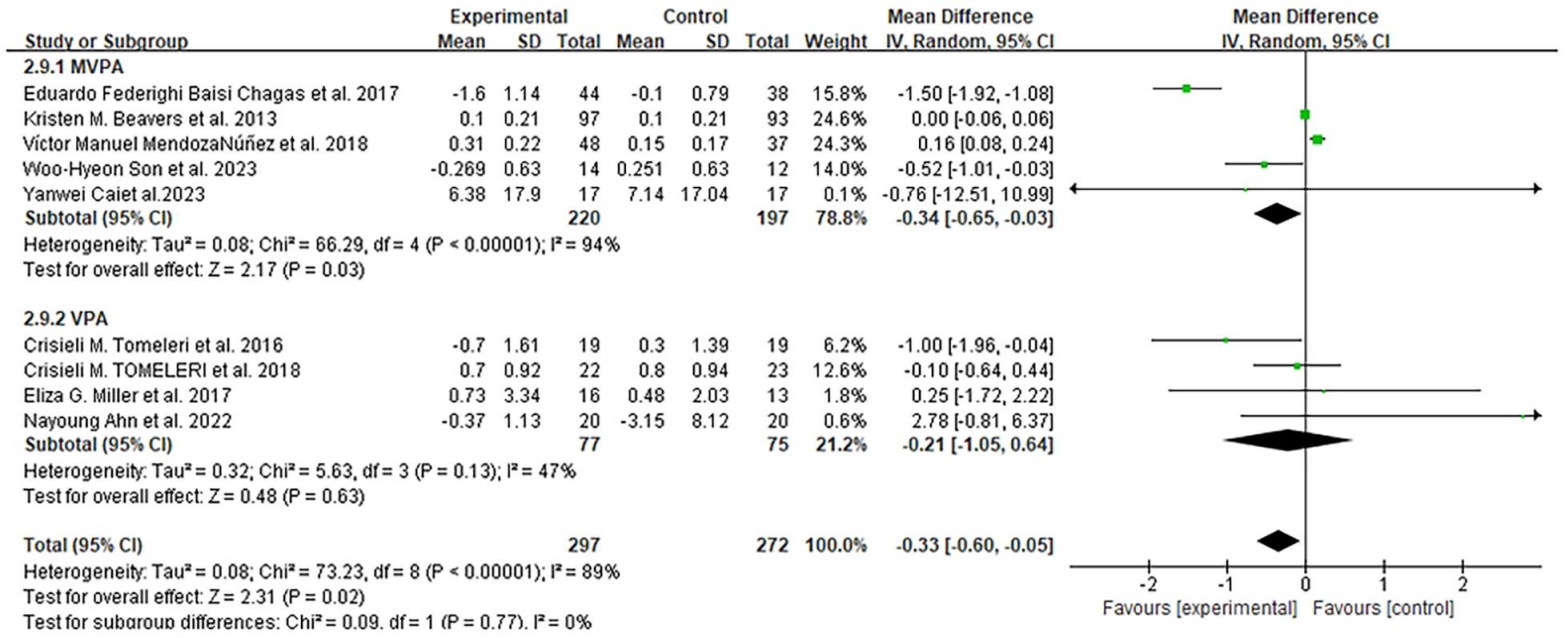
Forest plots of the effect of physical activity on IL-6 compared with no exercise.

Nine studies assessed IL-6 levels at different time points (8 weeks to 18 months) (47, 48, 54, 56, 58–62). Meta-analysis showed that exercise significantly reduced IL-6 levels in older adults compared to the control group (MD = −0.33 pg/mL, 95% CI: −0.60 to 0.05, *I*^2^ = 89%, *p* = 0.02), with details presented in Figure 9.

Subgroup analysis by exercise intensity revealed that MVPA [five studies (48, 56, 59, 61, 62)] significantly reduced IL-6 by 0.34 pg/mL (95% CI: −0.65 to −0.03), while VPA [four studies (47, 56, 58, 60)] showed a non-significant reduction of 0.21 pg/mL (95% CI: −1.05 to 0.64). By intervention duration: five studies with <6 months of exercise (48, 56, 59, 61, 62) reported an IL-6 reduction of approximately 0.60 pg/mL (95% CI: −1.25 to 0.05, *p* = 0.07), while three studies with ≥6 months (47, 54, 58) showed a non-significant increase of 0.18 pg/mL (95% CI: −0.10 to 0.46, *p* = 0.21). By exercise modality: four aerobic interventions (47, 48, 59, 61) reduced IL-6 by 0.34 pg/mL (95% CI: −0.65 to −0.03); three RT studies (56, 58, 62) had a non-significant reduction of 0.24 pg/mL (95% CI: −1.31 to 0.83); and one combined training study (60) reported a reduction of 0.76 pg/mL (95% CI: −12.51 to 10.99).

By participant health status: exercise was associated with reduced IL-6 in obese and healthy older adults, but results were inconsistent in older adults with diabetes or MetS, with no significant differences observed.

#### 3.5.8 Additional cytokines and biomarkers

One study (58) found that dynamic resistance exercise significantly increased IL-4/IL-5 levels compared to controls. One study (46) reported no significant change in IL-1β level post-exercise. One study (64) noted a significant increase IL-10 level with exercise, two studies (46, 47) found no effect, and one study (54) reported a reduction with progressive RT. One study (48) on overweight/obese older adults showed that combined exercise and weight loss significantly lowered leptin levels, while exercise alone had no significant effect. Due to limited studies and data, meta-analyses were not conducted for these biomarkers. Further high-quality trials are needed to clarify their responses to exercise in older populations.

#### 3.5.9 Adverse events

None of the studies included in this review reported adverse events.

## 4 Discussion

This systematic review and meta-analysis included 19 studies involving 1,130 participants to evaluate the effects of exercise interventions on obesity-related indices and inflammatory markers in older adults. The main results showed that regular exercise (including aerobic exercise, resistance exercise, and combined exercise) exerted significant beneficial effects on abdominal obesity and inflammatory status, with consistent directionality to well-established evidence, but subgroup analyses further uncovered nuanced patterns that address gaps in prior literature. These subgroup-specific insights not only reinforce the clinical relevance of exercise for older adults but also provide actionable guidance for tailoring interventions to diverse populations and health goals.

As core indicators of obesity, WC and BMI are closely linked to systemic inflammatory state and overall health in older adults. Elevated inflammatory markers often amplify age-related disease risk (65). Central obesity, which is driven by abdominal fat accumulation, acts as a key trigger for persistent low-grade inflammation in this population (66), and this makes targeted exercise interventions particularly valuable. Our subgroup analysis highlighted that exercise efficacy varies substantially by comorbidity status. This detail is often overlooked in aggregated analyses but has critical implications for clinical prioritization: prioritizing obese older adults and those with MetS for exercise interventions may yield greater reductions in abdominal obesity, and this aligns with the need to address high-risk subgroups first. Additionally, the observed effects of exercise on BF% and VFA further support its role in modifying adipose tissue distribution. Adipose tissue distribution is a key mediator of inflammation (67–69). This finding aligns with two prior meta-analyses focusing on older adults (34, 70), and these studies collectively confirm that regulating obesity via exercise is a viable strategy to mitigate inflammation in this age group.

The results of this study further reveal the inhibitory effect of exercise on inflammatory factors. CRP is not only a non-specific marker of inflammation but also actively involved in CVD such as atherosclerosis and vascular inflammation, making it a strong predictor and risk factor for cardiovascular events. TNF-α is considered one of the earliest and most critical inflammatory mediators in the immune response process, while IL-6 exhibits pro-inflammatory and pyrogenic properties. Adiponectin levels are negatively correlated with the risk of type II diabetes and coronary artery disease due to their anti-diabetic, anti-atherosclerotic, and anti-inflammatory effects. These biomarkers are often used as outcome indicators for exercise interventions aimed at improving the health of older adults. We extend this evidence by further stratifying exercise intensity: our data show that VPA reduces TNF-α more effectively than MVPA providing actionable intensity guidance for high-risk subgroups. RT also emerged as a key modality: it reduced CRP more than aerobic exercise and independently improved BMI. This is consistent with a meta-analysis on RT with/without caloric restriction (71), which found that RT alone reduces visceral fat and that combining it with caloric restriction enhances effects. While our study lacked dietary data, this alignment confirms RT's independent role in fat metabolism, critical for older adults unable to sustain high-volume aerobic exercise. We further identified a temporal pattern unreported in prior meta-analyses: short-term interventions (<6 months) were more effective for reducing TNF-α and IL-6, while long-term interventions (≥6 months) better improved BMI and CRP. This suggests that intervention duration should be tailored to specific health goals, e.g., short-term VPA for rapid inflammation reduction, and long-term combined training for sustained BMI control.

While CRP, TNF-α, and IL-6 are non-specific markers, their relevance is amplified in older adults, which is the core focus of our study. These markers are strongly linked to age-related morbidity and mortality: elevated CRP predicts CVD onset in older adults (10), and TNF-α/IL-6 correlate with sarcopenia and disability (24). Our findings add clinical value by showing that exercise reduces these markers even in high-risk subgroups: for example, older adults with diabetes (51, 64) had a significant CRP reduction, a change associated lower CVD risk in this population (72). According to the results of our meta-analysis, not only aerobic exercise but also resistance exercise and combined training are effective in reducing obesity and anti-inflammation, and even more effective in populations with comorbidities such as MetS and diabetes. Our findings are consistent with the conclusions of two recent meta-analyses (35, 73). This suggests that personalized exercise programs need to combine multiple exercise modalities to maximize health benefits.

The results of this study are consistent with, but extend, previous systematic reviews and meta-analyses. Liang et al. (2021) (74) showed that combined aerobic-RT is most effective for weight loss and WC reduction, our data support this but add that VPA enhances TNF-α reduction and that duration modifies BMI outcomes. Fossati et al. (35) confirmed exercise's role in reducing pro-inflammatory cytokines in older adults, we build on this by showing that these effects are strongest in those with MetS or diabetes, and by linking cytokine reductions to specific exercise modalities (e.g., RT for CRP). In addition, existing literature has shown a negative correlation between physical activity levels and CRP levels in older adults (75), which is consistent with the results of this study. It is worth noting that the association between exercise, abdominal obesity, and inflammatory markers appears to be more significant in men than in women (76). A review by Cronin et al. pointed out that although the effects of aerobic and RT on CRP and IL-6 in adults are inconclusive, exercise interventions in the elderly population (>65 years old) tend to produce more significant reductions in inflammatory biomarkers (77). In terms of anti-inflammatory cytokines, increased serum interleukin-10 (IL-10) levels are associated with a reduced risk of future cardiac events because it plays a role in inhibiting the occurrence of atherosclerosis and systemic inflammation, including inhibiting nuclear factor-κB activation, metalloproteinases, tissue factor expression, and apoptosis (72, 78). The above conclusions suggest that exercise may exert anti-inflammatory effects through two key mechanisms: one is to reduce visceral fat (the main source of pro-inflammatory cytokines such as TNF-α), and the other is to stimulate skeletal muscle to release anti-inflammatory myokines (such as IL-6 and IL-10), which inhibit pro-inflammatory signaling through pathways such as NF-κB (35, 79). This also explains why exercise can reduce the levels of CRP, TNF-α, and IL-6—these markers are closely related to CVD and mortality in older adults (9, 14, 23).

This analysis found no significant effect of exercise on adiponectin levels, which contradicts some early research results (80). This discrepancy may be due to the following reasons: the included population mostly consists of patients with obesity or MetS (with low baseline adiponectin levels), short intervention duration (<6 months), and uncontrolled confounding factors such as diet and medication. The regulation of adiponectin depends on long-term metabolic adaptation and sustained fat reduction (80, 81), which may not have been achieved in the included studies.

In conclusion, our review confirms exercise's positive effects on abdominal obesity and inflammation in older adults but goes beyond duplicating known data by providing subgroup-specific, actionable insights. By integrating findings from recent studies (35, 73) and standardizing exercise load reporting, we strengthen the evidence base for targeted exercise interventions—particularly for high-risk older adults with chronic conditions. Future research addressing dietary monitoring and long-term follow-up will further refine these recommendations and enhance our understanding of exercise's role in healthy aging.

## 5 Limitations and strengths

This meta-analysis has several limitations to consider when interpreting results. First, language bias is possible. Only English-language studies were included due to a lack of reviewers proficient in non-English languages, which may omit relevant non-English literature and limit findings' generalizability. Second, significant heterogeneity existed in some inflammatory markers, potentially from varied exercise protocols (intensity, type), participants' baseline health (obesity degree, disease control), and inconsistent outcome measurements. While subgroup (by intensity, duration, population) and sensitivity analyses explored heterogeneity sources, unreported factors (e.g., exercise adherence, baseline inflammatory levels) still might have affected effect size estimation. Third, standardized dietary data were lacking across all 19 RCTs. No study reported detailed diets or used tools like 24-h dietary recalls to monitor intake, making it impossible to separate exercise's independent effects from exercise and diet interactions. Unreported caloric restriction in some groups may have synergistically reduced visceral fat and inflammatory markers, overestimating exercise's independent effect. Fourth, classifying combined exercise was challenging. Among 8 combined training studies, exercise type proportions and intensity progression varied greatly, hindering accurate efficacy comparisons. Without standardized combined training definitions, subgroup findings' reproducibility may be limited. Finally, network meta-analysis was unfeasible. High heterogeneity in exercise programs (duration, intensity progression) and limited indirect comparisons between interventions prevented a fully connected network. Traditional pairwise meta-analysis was used instead, limiting the ability to rank different exercise types' relative efficacy for specific outcomes.

The strengths of this study are as follows: it further clarified the specific effects of exercises of different intensities and modalities in diverse elderly populations (including those with chronic diseases), expanding the depth of existing research. Secondly, it comprehensively evaluated the impact of exercise on obesity-related indices and inflammatory factors in healthy older adults and those with metabolic diseases such as obesity, diabetes, and MetS. By systematically analyzing the effects of three intervention types (aerobic exercise, RT, and combined training) and two exercise intensities (MVPA and VPA), it revealed multiple mechanisms by which exercise regulates obesity and chronic inflammation. Finally, it quantitatively synthesized key indicators such as WC, BMI, and inflammatory markers, providing a solid evidence base for clinicians and researchers interested in optimizing physical activity interventions for the elderly population.

## 6 Conclusion

This systematic review and meta-analysis confirm that regular exercise is a safe, non-pharmacological intervention for improving abdominal obesity and inflammatory status in older adults, with distinct subgroup-specific effects. Exercise interventions (aerobic, resistance, and combined training) can significantly reduce obesity indicators including WC, BMI, and BF%, while lowering levels of inflammatory markers such as CRP, TNF-α, and IL-6. However, no significant effect on adiponectin was observed, which may be related to limited data and baseline differences. Subgroup analyses revealed that: older adults with obesity or MetS derived the greatest benefits; moderate-to-high intensity combined aerobic-RT (3–5 sessions/week, 30–60 min/session, ≥12 weeks duration) was the most effective, with VPA showing superior TNF-α-lowering effects compared to MVPA; short-term interventions (<6 months) were more suitable for inflammation control, while long-term interventions (≥6 months) exhibited more sustained BMI improvement. This study has limitations such as the lack of dietary data. Future research should incorporate standardized dietary monitoring, precise abdominal fat measurement via CT, and long-term follow-up. In clinical practice, customizing exercise programs for different populations can maximize health benefits, and subsequent studies will further refine these intervention recommendations.

## Data Availability

The original contributions presented in the study are included in the article/Supplementary Material, further inquiries can be directed to the corresponding author.
