# Supplementary material for: Effect of exercise on abdominal obesity and inflammatory response in the older adults: a systematic review and meta-analysis of randomized controlled trials
Source: Front Sports Act Living. 2026 Jan 6;7:1677087. doi: 10.3389/fspor.2025.1677087 (PMC12815447; doi:10.3389/fspor.2025.1677087)
Supplement: Supplementary file 1 [file Table1.docx]

**Table S1:** Search from and corresponding results

| **Data source** | **Search query** | **Results** |
| --- | --- | --- |
| **Web of science** | ((TS= older OR elderly age)) AND (TS= (aerobic exercise OR exercise OR physical activity OR Strength training OR comprehensive training)) AND (TS= (inflammation OR cytokines OR interleukin)) NOT (TS= (Review OR Overview OR Meta)) | 2080 |
| **Pubmed** | (“older” or “elderly” or “aged” or “elder” or “geriatric” or “elderly people” or “old people” or “old people” or “senior”) AND ("aerobic exercise" or "exercise" or "fast walking" or "tai ji" or "yoga" or "brisk walking" or "jogging" or "taijiquan" or "tai chi" or "qigong" or "physical activity" or "physical exercise" or "physical endurance" or "treadmill" or "bicycling" or "dance" or "rope skipping" or "resistance training" or "strength training" or "weight training" or "resistance exercise" or "combined exercise") AND (“inflammation” or “cytokines” or “interleukin” or “Tumor Necrosis Factor” or “Transforming growth factor” or “C-Reactive protein” or “IL-1“ or ”IL-6“ or ”IL-8“ or ”TNF-α“ or ”TGF-β“ or ”CRP“or “Obesity” or “abdominal obesity” or “metabolic syndrome”.) | 1141 |
| **CINAHL** | TX (aerobic exercise OR exercise OR physical activity OR physical exercise OR resistance training OR strength training OR combined exercise) AND TX (older OR elderly OR aged OR elder OR old people OR old people or senior) AND TX (inflammation OR cytokines OR interleukin) NOT TX ( Review OR Overview OR Meta ) | 50 |
| **Embase** | TX (aerobic exercise OR exercise OR physical activity OR physical exercise OR resistance training OR strength training OR combined exercise) AND TX (older OR elderly OR aged OR elder OR old people OR old people or senior) AND TX (inflammation OR cytokines OR interleukin) NOT TX ( Review OR Overview OR Meta ) | 1500 |
| **Cochrane Library** | Title Abstract keyword:( (“older” or “elderly” or “aged” or “elder” or “geriatric” or “elderly people” or “old people” or “old people” or “senior”) AND ("aerobic exercise" or "exercise" or "fast walking" or "tai ji" or "yoga" or "brisk walking" or "jogging" or "taijiquan" or "tai chi" or "qigong" or "physical activity" or "physical exercise" or "physical endurance" or "treadmill" or "bicycling" or "dance" or "rope skipping" or "resistance training" or "strength training" or "weight training" or "resistance exercise" or "combined exercise") AND (“inflammation” or “cytokines” or “interleukin” or “Tumor Necrosis Factor” or “Transforming growth factor” or “C-Reactive protein” or “IL-1“ or ”IL-6“ or ”IL-8“ or ”TNF-α“ or ”TGF-β“ or ”CRP“or “Obesity” or “abdominal obesity” or “metabolic syndrome”.) | 1335 |
| **Scopus** | (ALL (aerobic exercise OR exercise OR physical activity OR physical exercise OR resistance training OR strength training OR combined exercise) AND ALL (older OR elderly OR aged OR elder OR old people OR old people or senior) AND ALL (inflammation OR cytokines OR interleukin) AND NOT TITLE-ABS-KEY ( Review OR Overview OR Meta ) ) | 1516 |
