# Supplementary material for: Effect of exercise on abdominal obesity and inflammatory response in the older adults: a systematic review and meta-analysis of randomized controlled trials
Source: Front Sports Act Living. 2026 Jan 6;7:1677087. doi: 10.3389/fspor.2025.1677087 (PMC12815447; doi:10.3389/fspor.2025.1677087)
Supplement: Supplementary file 2 [file Table2.docx]

**Table S2: MET Values Conversion of Included Exercise Interventions**

| **Author, Year** | **Exercise Type** | **Original Exercise Description** | **MET Value Calculation** | **Weekly Total MET-min** |
| --- | --- | --- | --- | --- |
| Takahashi M;2013 | Aerobic | Walking 30-60 min/session, 5 sessions/week, ≥3MET | Walking: 3.5-5.9 MET（mean: 4.7 MET） | 4.7 MET × 45 min × 5 sessions = 1057 |
| Mendoza-Núñez VM; 2018 | Tai Chi | Tai Chi, 55 min/session,5 times/week. | 4 MET | 4 MET × 55 min × 5 sessions = 1100 |
| Beavers KM; 2013 | Aerobic | Walking 3-5 times/week, 150min/week, 13 on the Borg Scale. | Walking: 4.5 MET | 4.5 MET × 150 = 675 |
| Bouchonville M; 2013 | Multicomponent exercise sessions | Aerobic, resistance training and balance exercises; 90 min/session, 3 sessions/week, HR 65-85% max | Multicomponent exercise sessions 5-7 MET（mean: 6 MET） | 6 MET × 90 min × 3 sessions = 1620 |
| Gargallo P; 2024 | Multicomponent training or Power training | 60 min/session, 2 times/week, Borg 11-15. | 6 MET | 6 MET × 60 min ×2 sessions = 720 |
| Mavros Y; 2014 | Progressive resistance training | 3 times/week, 40-60min, 80%(1RM) or Borg 15-18. | 5-7 MET（mean: 6 MET） | 6 MET × 50 min × 3 sessions = 900 |
| Sardeli AV; 2022 | Multicomponent exercise sessions | Aerobic and resistance training; 55 min/session, 3 sessions/week, 63% maximum VO2max. | 4.5 MET | 4.5 MET × 55 min ×3 sessions = 742.5 |
| Kadoglou NP; 2007 | Aerobic | Walking 60 min/session, 4 sessions/week, (50–75%, VO2 peak) | Walking: 3.5-5.9 MET（mean:4.7 MET） | 4.7 MET × 60 min × 5 sessions = 1410 |
| Christensen P; 2013 | Functional weight-bearing exercises | Both light and more vigorous activities, 60 min/session, 4 sessions/week, 4-6 MET. | 4-6 MET(mean:5 MET） | 5 MET × 60 min × 4 sessions =1200 |
| Miller EG; 2017 | Progressive resistance training | 3 times/week, 40-60min, 75-85% (1RM) | 4.8-5.6 MET（mean: 5.2 MET） | 5.2 MET × 50 min × 3 sessions = 780 |
| Hasegawa N; 2017 | Aerobic | Cycling ergometer 55 min/session, 3 sessions/week, 40%-70% VO2 peak | 3-6 MET（mean: 4.5 MET） | 4.5 MET × 55 min × 3 sessions = 742.5 |
| Tomeleri CM; 2017 | Progressive resistance training | 3 times/week, 40-60min, 1RM. | 6 MET | 6 MET × 50 min ×3 sessions = 900 |
| Da Silva MAR; 2020 | Progressive resistance training | 3 times/week, 50min, 1RM. | 6 MET | 6 MET × 50 min ×3 sessions = 900 |
| Ahn N; 2022 | Dynamic-resistance exercise | 3 times/week, 20-40min, Borg 12-13. | 3-5.9 MET | 4.5 MET × 30 min ×3 sessions = 405 |
| Son WH; 2023 | Aerobic | Walking 50 min/session, 4 sessions/week, HR 65-85% max | Walking: 3.5-5.9 MET（mean: 4.7 MET） | 4.7 MET × 50 min × 5 sessions = 940 |
| Cai Y; 2023 | Yijinjing and Resistance exercise | 3-5 times/week; 68-76min/ session, 60%–70% HRmax | 4 MET | 4 MET × 70 min × 4 sessions = 1120 |
| Chagas EFB; 2017 | Aerobic | Walking 75 min/session, 3 sessions/week, (50–60%, VO2 peak) | Walking: 4-4.5 MET（mean:4.3 MET） | 4.3 MET × 75 min × 3 sessions = 967.5 |
| Tomeleri CM; 2016 | Progressive resistance training | 3 times/week, 45-50min/ session, 50-100% RM. | 4-6 MET | 5 MET × 50 min ×3 sessions = 750 |
| Azamian Jazi A; 2022 | Elastic bands resistance training | 3 times/week, 55min, Borg 12-14. | 4-6 MET | 5 MET × 55 min ×3 sessions = 825 |

**Notes：**

1. For MET value conversion, priority is given to referencing the 2011 Compendium of Physical Activities (Ainsworth et al.). If a specific exercise type is not explicitly included, reference is made to exercises of similar intensity (e.g., "Tai Chi" is referenced to " Moderate and low-intensity aerobic exercise," and "resistance band training" is referenced to "moderate-intensity resistance exercise");

2. Weekly total MET-minutes = Single-exercise MET value × Single-exercise duration (minutes) × Weekly exercise frequency;

3. If MET values are already provided in the original studies, they are directly cited and labeled as "Original Study Reported." If only heart rate (HR) or rating of perceived exertion (RPE) is provided, conversion is performed according to the following standards:

HR 50–60% max / RPE 9–11 → Low intensity (<3.0 MET);

HR 60–75% max / RPE 12–14 → Moderate intensity (3.0–5.9 MET, MVPA);

HR >75% max / RPE 15–17 → High intensity (≥6.0 MET, VPA).
