# Supplementary material for: Effect of exercise on abdominal obesity and inflammatory response in the older adults: a systematic review and meta-analysis of randomized controlled trials
Source: Front Sports Act Living. 2026 Jan 6;7:1677087. doi: 10.3389/fspor.2025.1677087 (PMC12815447; doi:10.3389/fspor.2025.1677087)
Supplement: Supplementary file 3 [file Datasheet1.docx]

**supplementary Figures S1-10**

**Subgroup analyses by disease type and exercise type**


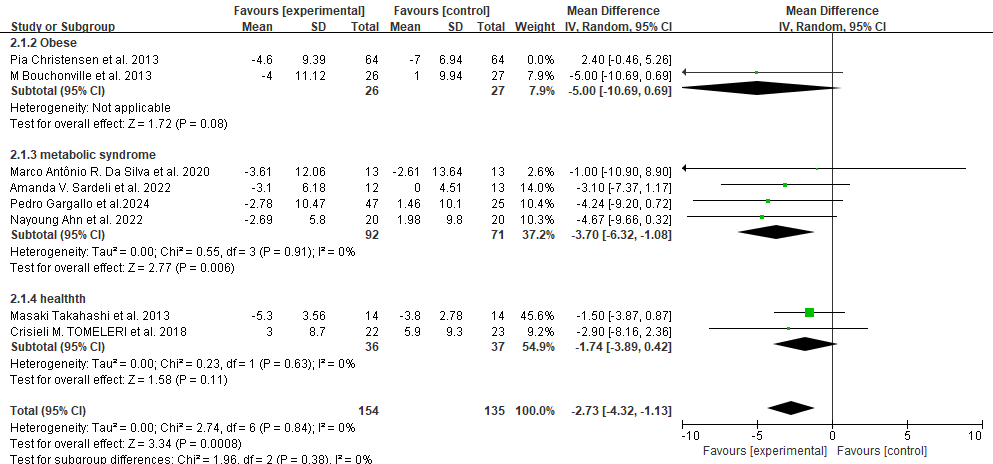


**Figure S1.** Forest plots of the effect of physical activity on WC in older adults with different disease types compared to no exercise


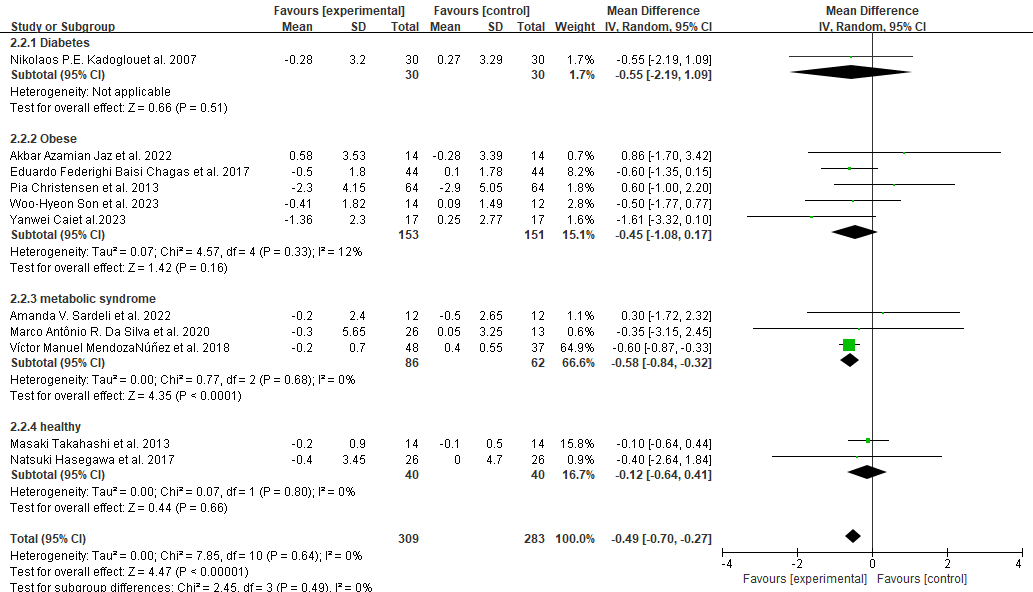


**Figure S2.** Forest plots of the effect of physical activity on BMI in older adults with different disease types compared to no exercise


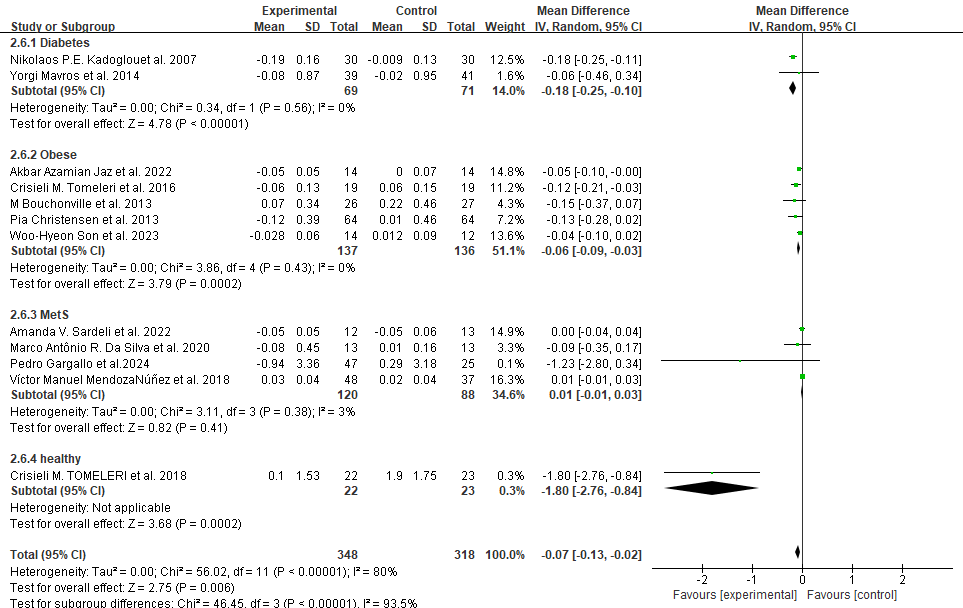


**Figure S3.** Forest plots of the effect of physical activity on CRP in older adults with different disease types compared to no exercise


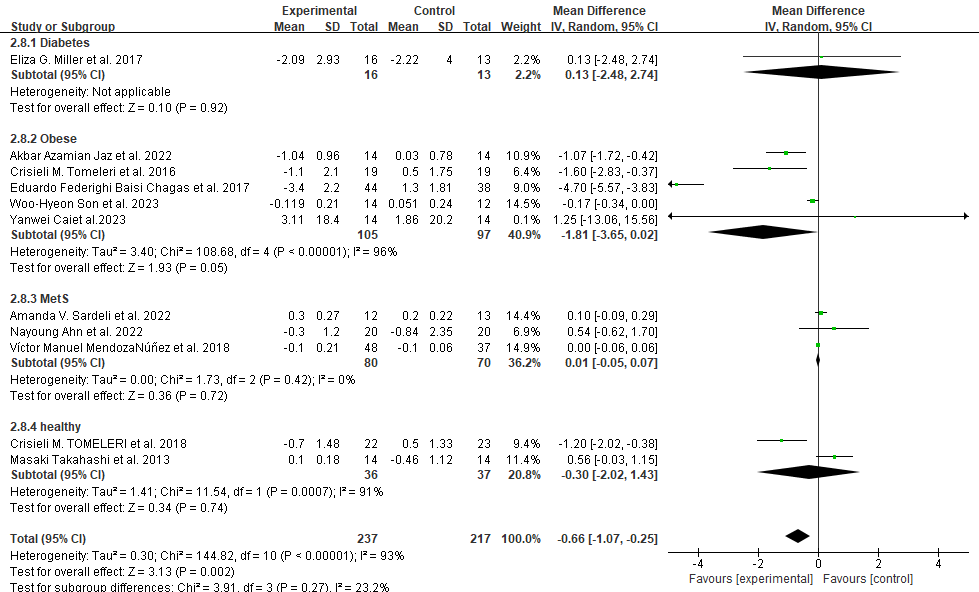


**Figure S4.** Forest plots of the effect of physical activity on TNF-α in older adults with different disease types compared to no exercise


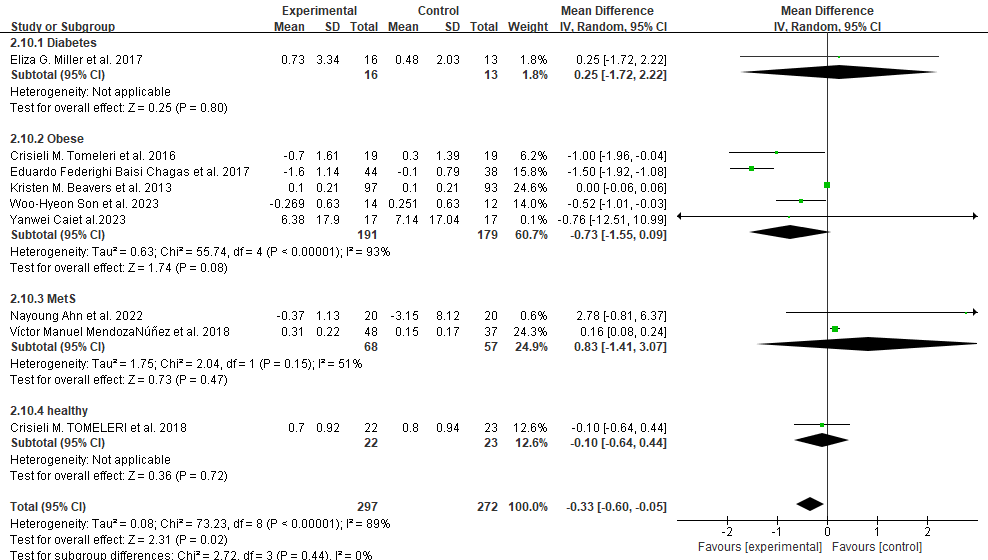


**Figure S5.** Forest plots of the effect of physical activity on IL-6 in older adults with different disease types compared to no exercise


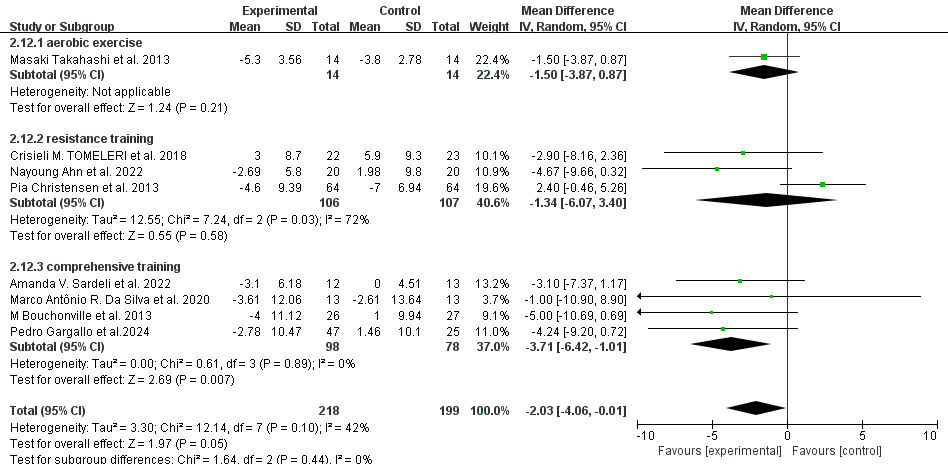


**Figure S6.** Forest plots of the effect of physical activity type on WC compared with no exercise


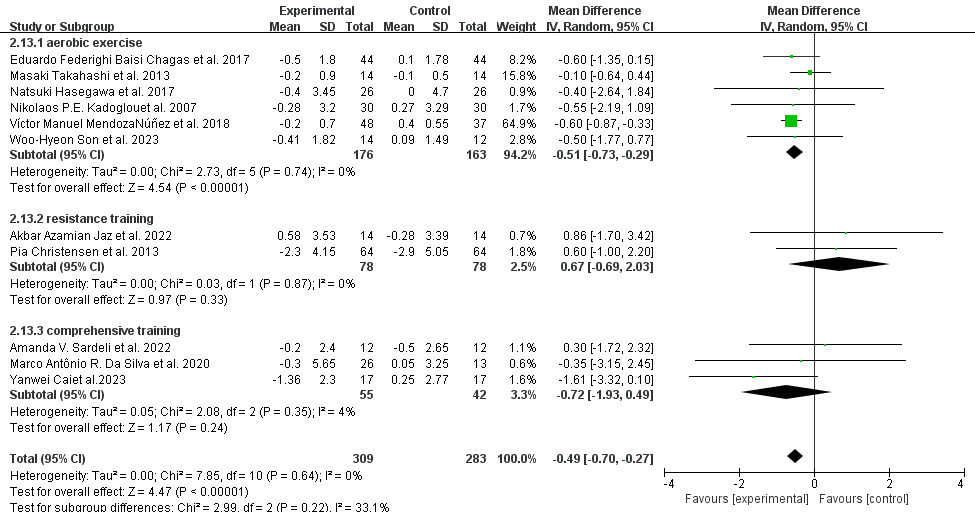


**Figure S7.** Forest plots of the effect of physical activity type on BMI compared with no exercise


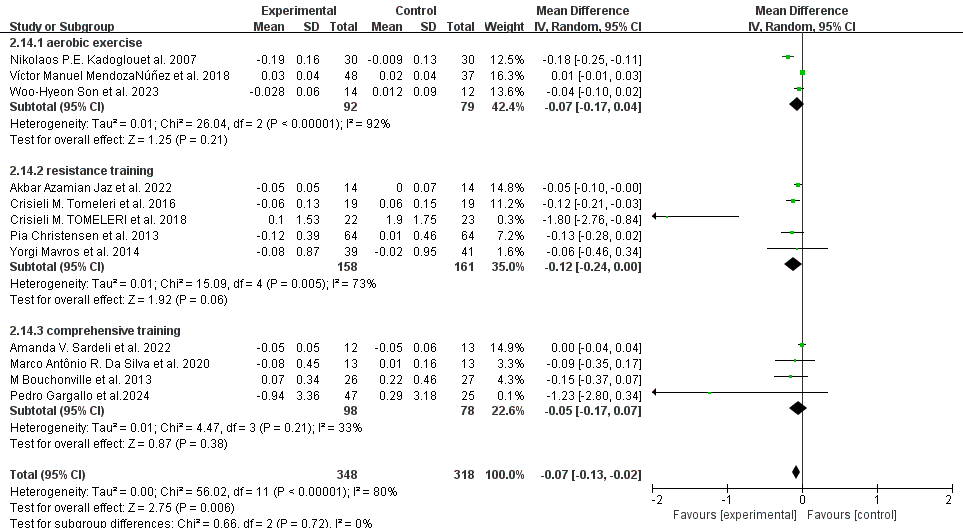


**Figure S8.** Forest plots of the effect of physical activity type on CRP compared with no exercise


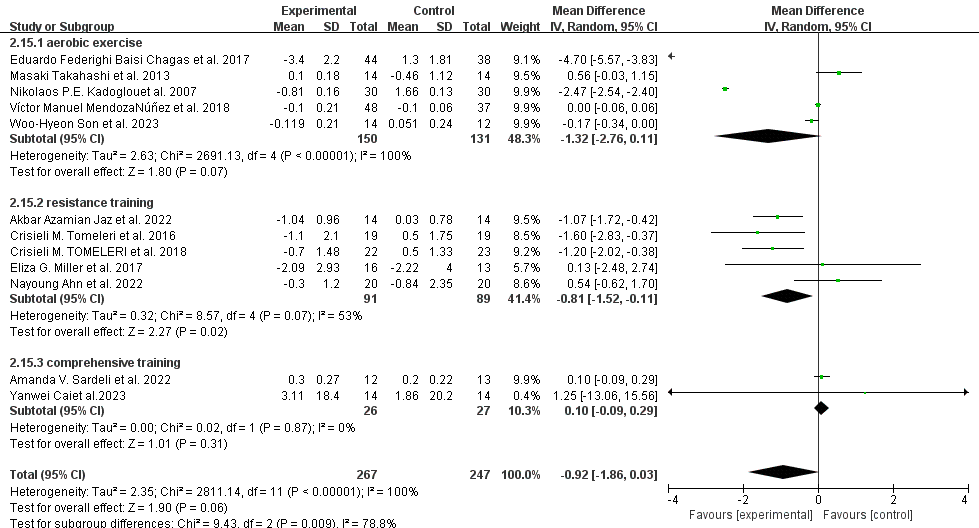


**Figure S9.** Forest plots of the effect of physical activity type on TNF-a compared with no exercise


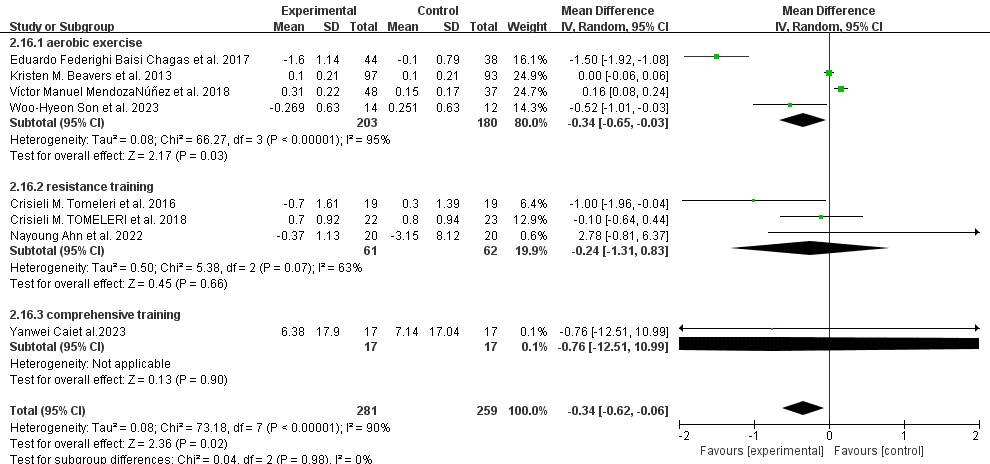


**Figure S10.** Forest plots of the effect of physical activity type on IL-6 compared with no exercise
